# Oncologic and Functional Outcomes of T3 Glottic Squamous Cell Carcinoma Following Transoral Laser Microsurgery: A Retrospective Chart Review

**DOI:** 10.1177/19160216251385930

**Published:** 2025-10-23

**Authors:** Victoria Taylor, Emma Bogner, Jessica Henley, Colin MacKay, Matthew H. Rigby, Martin Corsten, Jonathan Trites, Mark Taylor

**Affiliations:** 1 Faculty of Medicine, Dalhousie University, Halifax, NS, Canada; 2 Division of Otolaryngology—Head & Neck Surgery, Dalhousie University, Halifax, NS, Canada; 3 Division of Otolaryngology—Head & Neck Surgery, University of Calgary, Calgary, AB, Canada; 4 AdventHealth Medical Group, Division of Otolaryngology—Head & Neck Surgery, Daytona Beach, FL, USA

**Keywords:** transoral laser microsurgery, glottic cancer, laryngeal cancer

## Abstract

**Importance:**

Few centers globally use transoral laser microsurgery (TLM) for primary treatment of T3 glottic squamous cell carcinoma (SCC); consequently, data on its use in this setting are sparse.

**Objective:**

The aim of this study was to evaluate the oncologic and functional outcomes in T3 glottic SCC following TLM, including the impact of anterior commissure (AC) involvement.

**Design:**

Retrospective chart review.

**Setting:**

Single Canadian tertiary care hospital in Halifax, Nova Scotia, from January 2006 to December 2021.

**Participants:**

Adult (>18 years old) patients with T3 glottic SCC treated with TLM. Patients were excluded if they were previously treated for laryngeal cancer.

**Intervention or Exposures:**

This study used prospectively collected data for patients treated with TLM for T3 glottic SCC.

**Main Outcome Measures:**

Oncologic outcomes were assessed using disease-specific survival (DSS), local control (LC), and laryngectomy-free survival (LFS). Functional outcomes were measured using the Voice Handicap Index-10 (VHI-10).

**Results:**

In total, 28 patients (mean age = 68.4 years) underwent curative TLM for T3 glottic SCC. Sixteen of the cases involved the AC and 22 had paraglottic space involvement. Two and 5-year DSS were 79.2% (CI = 62.3%-100%). Two and 5-year LC were 80.2% (CI = 65.9%-97.4%) and 52.6% (CI = 27.4%-100%), respectively, and 2- and 5-year LFS rates were 86.1% (CI = 72.6%-100%) and 64.6% (CI = 35.8%-100%). AC involvement had no significant impact on DSS, LC, or LFS. There was no significant difference in preoperative and 3-month VHI-10 scores (*P* = .6632); however, there was significant improvement noted at the 6 (*P* = .042) and 12-month (*P* = .037) periods.

**Conclusions:**

TLM is a viable surgical option for appropriately selected patients with T3 glottic SCC, with or without AC involvement, achieving favorable oncologic and functional outcomes.

**Relevance:**

These findings further contribute to the limited evidence supporting the use of TLM in the management of advanced-stage glottic cancer.

## Graphical abstract

**Graphical abstract:**
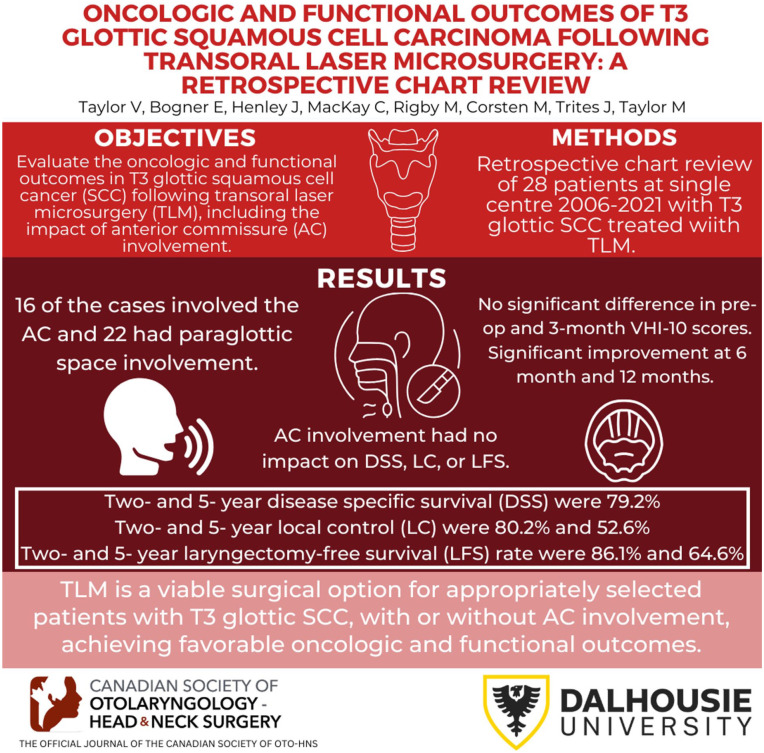

## Key Messages

- Transoral laser microsurgery (TLM) is an effective treatment modality for glottic squamous cell carcinoma (SCC), with less associated morbidity compared to open surgical techniques, radiotherapy, and chemoradiotherapy.
- Global adaptation of TLM for T3 glottic SCC remains limited, despite growing evidence supporting its use in this setting.
- Treatment decisions must balance patient values with survival outcomes.

## Introduction

Laryngeal cancer is the most prevalent malignancy of the head and neck, with glottic squamous cell carcinomas (SCC) representing its most common subtype.^1^ Historically, treatments for laryngeal SCC have included radiotherapy (RT), chemoradiotherapy (CRT), and open surgeries such as partial resection or total laryngectomy.^2,3^ In 1972, transoral laser microsurgery (TLM) was introduced as an alternative and less invasive treatment approach for laryngeal malignancies.^4^ Specifically, TLM has been found to provide comparable oncologic results to partial or total laryngectomy for T3 glottic cancer.^5,6^ Since the introduction of TLM, studies have demonstrated reduced morbidity, shorter recovery times, broader patient eligibility, and a reduction in post-surgical complications when compared to open surgical techniques.^2,7^

The QEII Health Sciences Centre (Halifax, NS, USA) has utilized TLM as an appropriate treatment for T3 glottic SCC for over 15 years. Previous data from our center have demonstrated that patients receiving TLM for T3 laryngeal cancer have equal or better outcomes when compared to RT or CRT.^8^ Unfortunately, considering the majority of evidence for TLM is geared toward early stage glottic cancer (T1-T2), and there is limited high-quality evidence for its use in T3 disease, global adoption of TLM for T3 glottic SCC remains low. Importantly, the appropriate selection of patients is essential. The purpose of this study is to provide an update of our experience with TLM in the management of T3 glottic SCC; specifically, we aimed to assess both oncologic and functional outcomes. In addition, given recent studies suggesting that anterior commissure (AC) involvement impacts outcomes in early stage glottic SCC, we sought to evaluate whether AC involvement influences oncologic or functional outcomes in T3 glottic SCC.^9^

## Materials and Methods

The study received research ethics board approval through the Nova Scotia Health Research Ethics Board (ROMEO #1020643).

### Design and Participants

A retrospective chart review of prospectively collected data was performed for patients undergoing TLM for T3 glottic SCC at the QEII Health Sciences Centre from January 2006 to December 2021. Informed consent was waived, given the retrospective nature of the study. Adult (>18 years old) patients with T3 glottic SCC who underwent TLM with curative intent were included in the study. Selection for curative intent TLM was determined through imaging, clinical visualization of the tumor with nasopharyngoscopy, and discussions with a multidisciplinary cancer care team. Favorable criteria for TLM included adequate laryngeal exposure during endoscopy, mobile as opposed to fixed arytenoids, limited pre-epiglottic and paraglottic space involvement, and absence of cartilage invasion.^10,11^ Patients were excluded if they had previously been treated for laryngeal cancer or if there was insufficient data available to allow for adequate review of functional and oncologic outcomes. TLMs were performed by 2 surgeons, both of whom completed fellowship training in head and neck surgical oncology.

### Outcomes and Measures

Patient demographics, including age, sex, smoking and alcohol history, initial staging, AC involvement, adjuvant CRT or RT, and length of follow-up, were collected. Oncologic outcomes were evaluated using 2- and 5-year overall survival (OS), disease-specific survival (DSS), local control (LC), and laryngectomy-free survival (LFS).

Functional outcomes were measured using the Voice Handicap Index-10 (VHI-10), which is a 10-part subjective questionnaire measuring the influence of voice quality on patient quality of life. The VHI-10 was administered preoperatively, as well as at 3, 6, and 12 months.

### Statistical Analysis

R Statistical Software (R Core Team 2022; version 4.2.2) was used for data analysis. Descriptive statistics were used to summarize patient demographics. *T*-tests and Fisher’s exact tests were used to compare demographic information between patients with and without AC involvement. AC involvement was binary without subcategorization. Age, length of follow-up, and time to recurrence were continuous variables, whereas smoking history, initial staging, adjuvant therapy, and need for laryngectomy were categorical variables. Oncologic outcomes, including OS, DSS, LC, and LFS, were calculated using Kaplan-Meier curves. Log-rank tests were used to compare survival outcomes between groups. *T*-tests were used to compare VHI-10 scores between time periods.

## Results

### Patient Demographics

In total, 28 patients were included in the study. The mean age of patients at treatment was 68.4 years (range 44-82, SD = 10.7). Twenty-four (85.7%) patients were male, and 4 (14.3%) were female. Mean length of follow-up was 29.8 months (range 0-179, SD = 36.1). Sixteen (57.1%) patients had AC involvement. Stage justification was recorded and reasoning included paraglottic space involvement (n = 22), vocal cord fixation (n = 6), and involvement of the arytenoid (n = 2), piriform sinus (n = 1), and laryngeal cartilage (n = 1). Smoking status was available for 27 of the 28 patients. Twenty-five (92.6%) patients had a positive smoking history, and 2 (7.4%) patients were nonsmokers. Sixteen patients (64.0%) reported consuming <1 to 7 drinks of alcohol/week, 4 (16.0%) reported 7 to 14 drinks/week, 3 (12.0%) reported 15 to 12 drinks/week, and 2 (8.0%) reported >21 drinks/week (Table 1).

**Table 1.** Demographics.

Age, y, mean (range)	68.4 (44-82)
Sex, male (%)	24 (85.7%)
Smoking history (%)	
Nonsmoker	2 (7.4%)
Ex-smoker >10 pack-year history	11 (40.7%)
>10 pack-year history	11 (40.7%)
Ex-smoker with unknown history	3 (11.1%)
Missing data	1
Alcohol history (%), drinks/week	
<1-7	16 (64.0%)
7-14	4 (16.0%)
15-21	3 (12.0%)
>21	2 (8.0%)
Missing data	3
Follow-up, mo (SD; range)	29.8 (36.1; 0-179)
Anterior commissure involvement (%)	16 (57.1%)
Fixed cord (%)	6 (21.4%)
Positive margin (%)	6 (21.4%)

### Oncologic Outcomes

**Figure 1.**
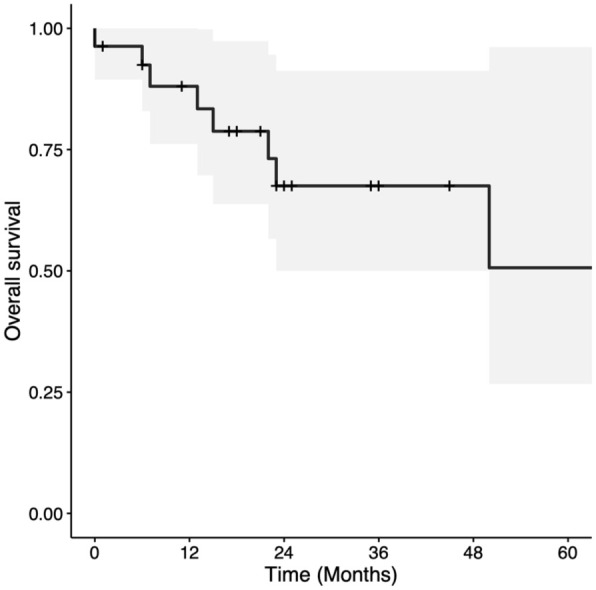
Kaplan-Meier curve for overall survival in patients with T3 glottic squamous cell carcinoma treated with transoral laser microsurgery.

**Figure 2.**
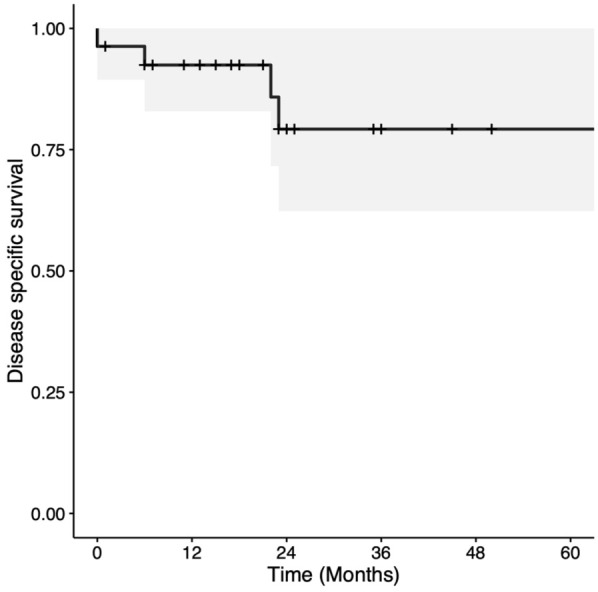
Kaplan-Meier curve for disease-specific survival in patients with T3 glottic squamous cell carcinoma treated with transoral laser microsurgery.

**Figure 3.**
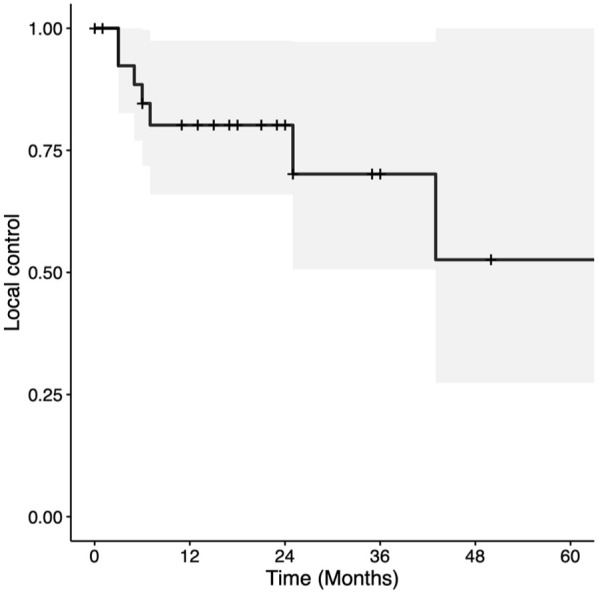
Kaplan-Meier curve for local control in patients with T3 glottic squamous cell carcinoma treated with transoral laser microsurgery.

**Figure 4.**
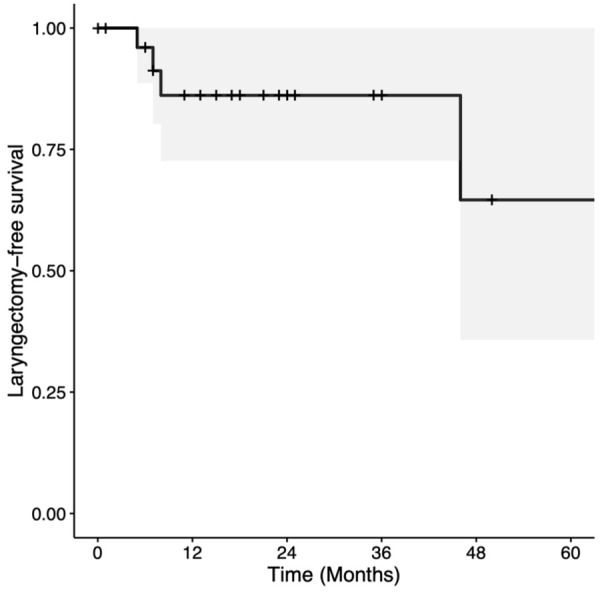
Kaplan-Meier curve for laryngectomy-free survival in patients with T3 glottic squamous cell carcinoma treated with transoral laser microsurgery.

Two and 5-year OS were 67.5% (CI = 50%-91.2%) and 50.6% (CI = 26.7%-96.1%), respectively (Figure 1). Two- and 5-year DSS were both 79.2% (CI = 62.3%-100%; Figure 2). Two- and 5-year LC rates were 80.2% (CI = 65.9%-97.4%) and 52.6% (CI = 27.4%-100%), respectively (Figure 3). In total, there were 7 (25.0%) local recurrences. Four (14.3%) patients required a total laryngectomy after primary management with TLM. Of the remaining 3 participants with local recurrences, 1 had a salvage TLM followed by adjuvant RT, 1 had salvage CRT, and 1 died of end-stage kidney disease 1 month after recurrence. This last patient was also tracheostomy and gastrostomy tube-dependent post-recurrence. Based on Kaplan-Meier estimates, the rates of 2 and 5-year LFS were 86.1% (CI = 72.6%-100%) and 64.6% (CI = 35.8%-100%), respectively (Figure 4). AC involvement had no statistically significant impact on LFS (*P* = .51), LC (*P* = .29), or DSS (*P* = .16). Conversely, OS was significantly lower for the participants without AC involvement (*P* = .039).

### Postoperative Complications

After surgery, 3 patients (10.7%) required a tracheostomy and 3 patients (10.7%) required a gastrostomy tube. As previously mentioned, 1 patient had both a tracheostomy and a gastrostomy tube following a local recurrence. One patient required a tracheostomy for airway obstruction secondary to a local recurrence, while another required a tracheostomy preoperatively due to anesthesia concerns, as the patient was on home oxygen. Of the remaining patients requiring gastrostomy tubes, 1 patient was gastrostomy tube-dependent preoperatively, and no details are available for the final patient. Thirteen patients (46.4%) required postoperative RT. Eight (28.6%) patients underwent adjuvant RT after the primary resection, 2 (7.1%) after a recurrence, and 3 (10.7%) after total laryngectomy.

### Functional Outcomes

**Figure 5.**
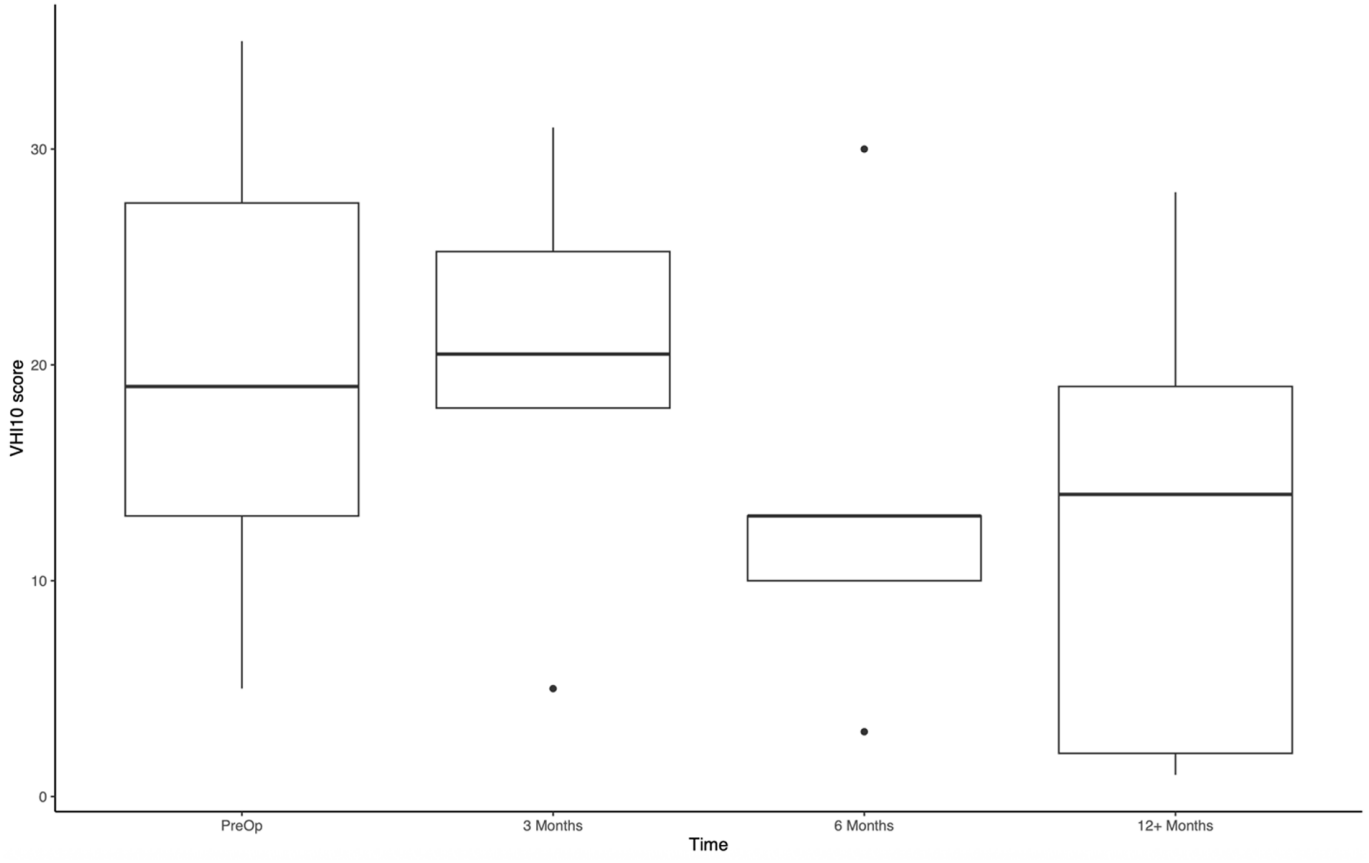
Voice Handicap Index-10 scores preoperatively and at 3, 6, and ≥12 months post-operatively.

Of the 28 patients included in the study, preoperative VHI-10 scores were available for 19 participants (Figure 5). Of those, 3-month scores were available for 7 patients, 6-month scores for 6 patients, and 12-month scores for 13 patients. Mean VHI-10 scores for the preoperative, 3, 6, and 12-month periods were 20.8, 20.2, 13.8, and 12.0, respectively. There was no significant difference in preoperative and 3-month VHI-10 scores (*P* = .6632). There was significant improvement noted at the 6- (*P* = .042) and 12-month (*P* = .037) periods, but the significance did not hold when adjusted for multiple tests. There was no significant difference in mean change in VHI-10 scores for patients with and without AC involvement from the preoperative period to 3 (*P* = .8497) or to 12 months (*P* = .7209). Due to insufficient sample size, *t*-tests were not performed to assess the preoperative to 6-month period.

## Discussion

### Comparison of Treatment Modalities

Management of advanced laryngeal tumors remains an area of ongoing debate. Much of the existing literature on TLM for glottic SCC focuses on early stage glottic SCC,^1^ including T1 to T2 disease, with limited data specific to T3 glottic SCC. TLM is a cost-effective and less invasive treatment modality compared to open surgeries, RT, and CRT, with equal or superior outcomes in early stage disease.^12^ Total laryngectomy achieves good oncologic outcomes but results in functional limitations and lower reported quality of life when compared to less invasive surgical strategies or CRT.^13^ Open partial laryngectomy has also been shown to be an effective treatment modality; however, it often results in less favorable functional outcomes when compared to TLM.^14^ RT alone has poorer oncologic outcomes than the surgical options mentioned. In a meta-analysis performed by Vaculik et al, TLM had favorable OS, DSS, and laryngeal preservation when compared to RT in treating early stage glottic cancer.^12^ A meta-analysis comparing treatment outcomes for T3 glottic SCC described lower LC rates with RT compared to total and partial laryngectomies, as well as higher laryngeal preservation rates for TLM and CRT when compared to RT alone.^5^

### Oncologic Outcomes

Several studies have evaluated the impact of TLM on oncologic outcomes in T3 glottic cancers. Peretti et al reported a 5-year OS and DFS of 65.7% and 72.9%, respectively, for 34 patients with T3 glottic cancers treated with TLM ± selective neck dissection ± adjuvant therapy.^15^ A Canadian study with 15 patients demonstrated a 2-year OS and DSS of 86.7% and 93.3%, respectively,^8^ and a larger study with 122 patients with T3 glottic SCC found 5-year OS, DSS, and LFS to be 58.6%, 84.1%, and 83%, respectively.^16^

The present study analyzed several oncologic outcomes, including OS, DSS, LC, and LFS. Two and 5-year OS rates were 67.5% and 50.6%, respectively—slightly lower than previously reported OS.^15,16^ Conversely, our study revealed 2- and 5-year DSS rates of 79.2%; comparable with values reported in the literature, with DSS rates ranging from 62% to 84%.^2,16,17^ The comparable rates of DSS and lower OS noted in the current study likely reflect a higher burden of non-oncologic mortality among the study population. Further studies may seek to explore comorbidity burden within the study population using the Charlson Comorbidity Index.

Two- and 5-year LC rates were 80.2% and 52.6%, respectively. The 2-year LC rates are comparable to similar studies, which report rates between 70% and 80%.^2,8^ In addition, Motta et al achieved a 5-year LC rate of 63% following TLM.^18^ The lower 5-year LC rate in this study may be attributed to the limited sample size. The notably wide confidence interval (CI = 27.4%-100%) may reflect the impact of the small number of patients to reach the 5-year endpoint. Given the typical course and presentation of patients with recurrent glottic cancers, patients who recurred would likely re-present for consideration of total laryngectomy (4/7 recurrences went on to have a total laryngectomy in this study). It is therefore possible that the true LC rate is closer to the raw recurrence rate of 75%.

LFS and/or laryngeal preservation are significant considerations in the management of T3 glottic SCC, and one of the advantages of treatment with TLM. In this study, 2- and 5-year LFS rates were 86.1% and 64.6%, respectively. This is consistent with another study from our institution, which reported a 2-year laryngeal preservation rate of 86.7% for patients with T3 glottic cancer treated with TLM.^8^ Studies from other centers report similar values.^17,18^

### Impact of AC Involvement

Numerous studies have highlighted the impact of AC involvement on treatment outcomes in glottic cancer. For example, in early glottic cancer, involvement of the AC has been associated with a poorer prognosis, voice outcomes, and increased risk of recurrence compared to glottic cancers without AC involvement.^19^ A study with 261 patients found that the group with grade 3 AC involvement, based on the Rucci classification system, and true subglottic extension, were associated with worse LC rates despite negative surgical margins confirmed by histological analysis.^20^ One of the largest studies examining AC involvement on early glottic cancer treated with TLM found no statistical difference in rates of local recurrence and laryngeal preservation between individuals with and without AC involvement.^21^

These results are comparable to the results of this study, where AC involvement did not significantly impact LFS, LC, or DSS. Other studies, however, have found a significant difference in LC rates in patients with extensive infiltration of the AC.^20,22^ These studies used Rucci’s classification system to subcategorize AC involvement based on extent. Those with extensive involvement of the AC on both sides of the midline showed worse outcomes for LC; however, this effect was not detected in all individuals with AC involvement. The current study demonstrated excellent oncologic outcomes despite AC involvement; however, it did not categorize AC involvement using Rucci’s classification. This may warrant further investigation in future studies.

Interestingly, this study also found that participants with no AC involvement had significantly lower rates of OS compared to those with AC involvement. This, however, is likely due to a higher burden of non-oncologic disease present in patients with no AC involvement, as there was no difference in DSS between groups. Comorbidity burden among the participants was not assessed; therefore, no absolute conclusions can be made about these results. Additionally, with a small sample size of 28 patients and even smaller numbers in each subgroup, survival analyses are underpowered. Subgroup comparisons, therefore, must be interpreted with caution.

### Functional Outcomes

This study found that voice outcomes, measured using the VHI-10 questionnaire, improved after TLM. A meta-analysis conducted by Cohen et al compared outcomes of TLM and RT in patients with T1 glottic cancer and revealed equivalent levels of voice handicap between the 2 treatment modalities.^23^ In addition, Chien et al distributed questionnaires to assess various quality of life measures, including voice quality post-TLM, and reported highly satisfactory voice outcomes following TLM.^17^ Although some patients experienced a mild decline in speech after TLM, the overall impact on voice-related quality of life was favorable. Furthermore, a few issues with swallowing, social eating, and social contact were reported upon questioning.^17^ A systematic review and meta-analysis conducted by Guimarães et al also reported no significant difference in self-assessment of voice quality for patients with early glottic cancer when treated with TLM compared to RT.^24^ Overall, the existing evidence suggests that TLM is associated with favorable functional outcomes in terms of voice-related quality of life in early glottic cancer, with only mild and manageable voice issues observed post-operatively; however, these results must be interpreted with caution in the setting of advanced glottic disease, considering there is a sparsity in studies focused on patients with T3 glottic cancer. Further research is warranted to better understand functional outcomes for advanced disease.

There is limited literature evaluating the effect of AC involvement on functional outcomes in glottic SCC. To our knowledge, there are no studies that have used the VHI-10 questionnaire as a measure of voice outcome in this group, as existing studies use more vague descriptions of voice quality. Blanch et al investigated voice outcomes after TLM in patients with glottic SCC with AC involvement and found that voice quality was normal or useful in 66.4% of patients.^25^ The current study did not find a difference in change in VHI-10 scores in patients with and without AC involvement at both the 3- and 12-month time periods. This finding suggests that AC involvement does not predict worse self-reported voice outcomes post-TLM, and that AC involvement may not have an impact on functional outcomes in patients with T3 glottic SCC.

### Limitations

This study has several limitations to note. First, the study design itself is susceptible to potential biases. Selection bias might have played a role as we excluded patients with limited data, which could have influenced the comparability of outcomes. Furthermore, the availability and completeness of data in the database and patient charts were limiting factors. Of note, the small sample size in our study posed challenges in assessing differences in oncologic and functional outcomes between patients with and without AC involvement. In addition, although the VHI-10 is a validated tool to assess functional outcomes, it relies on the subjective perception of voice quality. The use of additional tools to measure functional outcomes would have further strengthened the results of this study with respect to voice outcomes.

## Conclusion

There is an ongoing debate regarding the superior treatment modality for advanced-stage glottic cancer, with limited literature assessing the use of TLM for T3 glottic cancer, specifically. The findings of this study support the use of TLM as a viable surgical option for appropriately selected patients with T3 glottic cancer, with and without AC involvement. To encourage widespread use of TLM in this setting and to optimize patient selection, further research with larger sample sizes is required.
